# Salivary pH within multifactorial caries risk assessment in children: observational clinical evidence using the Cariogram platform

**DOI:** 10.3389/froh.2026.1822466

**Published:** 2026-04-21

**Authors:** Eliza Denisa Barbulescu, Corina Marilena Cristache, Elena Valentina Vacarel, Vanda Roxana Nimigean

**Affiliations:** 1 Doctoral School, “Carol Davila” University of Medicine and Pharmacy, Bucharest, Romania; 2 Department of Dental Techniques, Faculty of Midwifery and Nursing, “Carol Davila” University of Medicine and Pharmacy, Bucharest, Romania; 3 Department of Oral Rehabilitation, Faculty of Dentistry, “Carol Davila” University of Medicine and Pharmacy, Bucharest, Romania

**Keywords:** caries risk assessment, Cariogram, digital diagnostics, pediatric dentistry, preventive dentistry

## Abstract

**Introduction:**

Dental caries is a multifactorial disease influenced by biological, behavioral, and preventive factors. Salivary pH has been proposed as a non-invasive biomarker for caries risk assessment, however, its independent clinical relevance within structured digital risk models remains unclear. This study school-aged pediatric cohort using the Cariogram platform as a structured, multifactorial caries risk assessment tool, and to evaluate the role of digitally measured salivary pH in relation to caries risk stratification and clinical caries indicators within this framework.

**Methods:**

A total of 66 children aged 6–12 years were included. Unstimulated salivary pH was measured using both a digital pH meter and colorimetric strips. Caries risk was assessed using the Cariogram platform. Agreement between pH measurement methods was evaluated using Bland–Altman analysis. Associations between salivary pH, dietary intake frequency, and caries risk were explored using Spearman's rank correlation. A multivariable linear regression model was constructed using the Cariogram-derived chance to avoid new caries (%) as the dependent variable, including mean digitally measured salivary pH, dietary intake frequency, oral hygiene status, fluoride toothpaste use, age, gender, and saliva collection timing as predictors.

**Results:**

Digital salivary pH values ranged from 6.1 to 7.2, with a peak between 6.6 and 6.8. Strip-based measurements systematically underestimated pH, with a mean bias of approximately 0.24 pH units. Salivary pH showed weak or negligible associations with Cariogram risk categories. In the multivariable model, dietary intake frequency, oral hygiene status, fluoride toothpaste use, and saliva collection timing were significant predictors of caries risk, whereas salivary pH was not independently associated after adjustment for behavioral and preventive factors.

**Conclusions:**

Salivary pH is a biologically relevant but insufficient standalone marker of caries risk in children. Its clinical value emerges when measured reliably and interpreted within a multifactorial, caries risk-assessment framework supported by digital tools, particularly the Cariogram platform and digital pH measurement.

## 1 Introduction

Dental caries remains one of the most prevalent chronic diseases of childhood and continues to impose a substantial clinical and public-health burden despite advances in prevention (1). Contemporary cariology defines dental caries as a non-communicable disease resulting from complex interactions between behavioral, dietary, microbial, and host-related factors. The caries process is dynamic, involving alternating phases of demineralization and remineralization, with lesion initiation and progression determined by the balance between pathological influences, such as frequent intake of fermentable carbohydrates, the presence of an acidogenic and aciduric biofilm, and reduced salivary protective capacity. Conversely, protective factors—including adequate fluoride exposure, effective salivary buffering, and appropriate plaque control—contribute to the maintenance of mineral homeostasis and caries prevention (2).

In school-aged children, the mixed dentition stage introduces additional vulnerability: newly erupted permanent molars exhibit immature enamel and anatomically complex fissures, while behavioral patterns (dietary autonomy, irregular oral hygiene) can exacerbate risk. These realities support a preventive paradigm that prioritizes individualized risk stratification and tailored management in favor of uniform, procedure-centered care (3, 4).

Caries risk assessment (CRA) has therefore become a cornerstone of evidence-based pediatric dental care, enabling clinicians to identify high-risk patients, determine recall intervals, and implement risk-based preventive and non-operative interventions (5). Major frameworks—including the International Caries Classification and Management System (ICCMS™) (6) and Caries Management by Risk Assessment (CAMBRA) (7)—explicitly place patient-level risk determination at the start of the clinical decision pathway, linking risk status to lesion management and personalized prevention plans. Professional guidance for pediatric populations similarly emphasizes structured CRA using age-appropriate indicators spanning social/behavioral determinants, clinical disease indicators, and protective factors, reflecting the multifactorial nature of dental caries (8).

Despite broad acceptance of CRA as a core component of preventive dentistry, its consistent implementation in routine clinical practice remains challenging. Digital multivariate tools such as the freely available Cariogram platform integrate clinical, behavioral, and biological variables to provide a pragmatic and structured estimation of individual caries risk and have demonstrated utility in both research and clinical settings. However, recent literature emphasizes that the predictive performance of CRA models may be enhanced by refining and expanding the biological inputs included, particularly through the integration of salivary biomarkers, in order to improve risk discrimination and clinical relevance across pediatric populations (5).

Saliva is increasingly recognized as a biologically plausible and clinically accessible source of host-protection markers relevant to caries susceptibility. Saliva supports oral homeostasis through clearance of sugars, buffering and neutralization of acids, calcium/phosphate delivery for remineralization, antimicrobial activity, and modulation of biofilm ecology (9). Among salivary parameters, pH reflects the biochemical environment that influences enamel dissolution and microbial selection; repeated or prolonged acidification favors demineralization and promotes aciduric bacterial communities. While salivary pH should not be interpreted as a standalone diagnostic test for caries, growing evidence supports its relevance as one component within multifactorial risk models, particularly when considered alongside flow rate, buffering capacity, dietary exposures, fluoride, and clinical disease indicators (10).

Recent pediatric and school-based investigations further suggest that more acidic salivary profiles and impaired buffering may be associated with higher caries burden or caries risk, although effect sizes and significance vary depending on sampling methods (stimulated vs. unstimulated saliva), timing, and population characteristics (11–13).

Parallel to advances in cariology, dentistry is undergoing rapid digital transformation, with increasing adoption of electronic health records, chairside diagnostics, mobile technologies, and data-driven decision support. In caries care, digital CRA is particularly appealing because multifactorial inputs can be captured in a standardized format, scored automatically, and translated into actionable prevention pathways. Emerging evidence indicates that algorithmic and machine-learning approaches can support risk prediction, potentially enhance consistency and enable integration into digital workflows and population programs. Importantly, the clinical acceptability of such systems depends on transparent variables, feasibility in real-world settings, and alignment with established preventive guidelines (14).

A key enabling development is the increasing availability of digital salivary pH measurement, which may improve reproducibility and practicality compared with subjective chairside estimations, and may facilitate longitudinal monitoring. Recent work has specifically discussed the integration of digital pH meters into pediatric caries prevention pathways and explored how pH data could be combined with broader digital risk assessment and, potentially, artificial intelligence–supported decision-making (15). Such approaches are consistent with the broader movement toward precision prevention: using measurable biological and behavioral inputs to tailor preventive intensity, optimize resource allocation, and engage patients and caregivers with personalized feedback (16).

Against this background, the present observational clinical study focuses on children aged 6–12 years and examines salivary pH within an established multifactorial caries risk-assessment framework. Specifically, the study applies the Cariogram platform as a structured digital tool for risk stratification and evaluates how digitally measured salivary pH relates to caries risk classification and clinical caries indicators alongside established behavioral, clinical, and protective variables. The aims were to (i) characterize caries risk profiles in a school-aged pediatric cohort using the Cariogram platform, and (ii) assess the extent to which salivary pH contributes to risk stratification within this existing multifactorial framework. Generating clinically grounded evidence in this age group is particularly relevant because risk status can change rapidly during mixed dentition, and early identification of high-risk children enables timely, targeted preventive interventions consistent with contemporary guidelines (5, 17).

## 2 Materials and methods

This clinical study followed an observational, cross-sectional design in accordance with the Strengthening the Reporting of Observational Studies in Epidemiology (STROBE) Statement and was conducted between May 2025 and January 2026 in a dental clinic affiliated to “Carol Davila” University of Medicine and Pharmacy from Bucharest, Romania. The investigated population consisted of children attending routine dental consultations and treatments, in an urban pediatric dental setting.

The research adhered to the Declaration of Helsinki, and was approved by the Ethics Committee of “Carol Davila” University of Medicine and Pharmacy of Bucharest, Romania (Approval No. 7629/04.04.2025). Written informed consent was obtained from parents or legal guardians, and the procedures were explained to each child in age-appropriate language.

Clinically healthy children aged 6–12 years were included. Patient selection was consecutive among those scheduled for prophylaxis (scaling, professional brushing, air-flow polishing, fluoride application), restorative treatment, or planned extractions where indicated. Inclusion criteria comprised good general health without known systemic disease, adequate cooperation during sample collection. No eligible participants declined participation or were excluded after enrollment due to incomplete data, and all enrolled participants were included in the final analysis.

Exclusion criteria were: systemic diseases or medications with the potential to alter salivary flow; salivary gland disorders; autism spectrum disorders (deliberately excluded to control cooperation and standardize sampling); acute oral infections or conditions that could contaminate the sample; and failure to comply with pre-analytical restrictions (recent food intake or oral hygiene procedures).

Given that salivary pH is sensitive to external factors, strict control of pre-analytical variables was implemented to improve comparability among participants. Standardization included:

Pre-collection restrictions: Children were instructed not to eat or brush their teeth for at least 1 h before the appointment, to avoid sweetened or carbonated beverages near the sampling time, and to consume only water, when necessary, with sufficient time allowed to minimize immediate dilution effects.

Sampling time and circadian control: The time of saliva collection was recorded and coded as a.m. (08:00–13:00) or p.m. (14:00–19:00).

Avoidance of procedural influence: Saliva collection was performed prior to any dental intervention to eliminate effects related to irrigation, materials, micro-bleeding, or immediate procedural stress.

Unstimulated saliva samples were collected to reflect the basal condition of the oral cavity. Using a passive expectoration technique into a sterile container over an approximately 30-minute pre-appointment interval, and without mechanical (chewing gum) or chemical (citric acid) stimulation, a volume of approximately 15–20 mL was obtained, adapted to the child's tolerance and clinical time constraints. Samples were processed immediately for pH determination, and prolonged exposure to air was avoided to reduce variations due to carbon dioxide release.

### 2.1 Salivary pH measurement: instruments, calibration, and procedures

#### 2.1.1 Digital method (pH meter)

A multifunctional digital pH meter (model EZ-9901/986, pH/TDS/EC/TEMP, Noyafa, Hong Kong, SAR China) with ±0.01 accuracy and automatic temperature compensation was used. The device was calibrated at the start of each session using three buffer solutions (pH 4.01; 6.86; and 9.18 at 25 °C). The manufacturer's recommended sequence was followed, and reading stability was verified before validating each calibration point. The electrode was rinsed with distilled water and gently dried between buffers to prevent cross-contamination, then immersed in the saliva sample. Stabilization time between determinations was 20–30 s. Three consecutive measurements were performed per sample, and the arithmetic mean was calculated as the “mean digital pH.” All values were recorded in a dedicated Excel database along with the other clinical variables.

#### 2.1.2 Colorimetric method (GC saliva check buffer strips)

GC Saliva Check Buffer (GC Europe A.G., Leuven, Belgium) strips (pH range 5.0–7.8) were used, with visual interpretation according to the manufacturer's colorimetric chart. The procedure reproduced typical chairside clinical use while documenting all steps to highlight potential error sources. The operational sequence was as follows: the strip was immersed in the same saliva sample for approximately 1 min, subsequently placed for a few seconds onto the pH meter sensor to ensure uniform contact and initial reaction. Two readings were recorded:

- the pH value displayed at the moment of strip contact with the sensor (two decimals), and
- the visually estimated pH after ∼30 s to 1 min (one decimal) according to the chart.

For comparison with the digital method, an arithmetic mean of the two strip-derived readings (strip–sensor + strip–visual) was calculated for each patient, yielding a representative indicator of the subjective method (“mean strip pH”).

This composite strip-based value was not intended to represent a validated reference standard, but rather to summarize the variability inherent in practical strip-based chairside assessment. It was used descriptively for method comparison, whereas only the mean digitally measured pH was entered into the multivariable model because it provided the more precise and reproducible continuous measure.

### 2.2 Caries risk assessment using the Cariogram platform

Caries risk assessment was performed for each participant using the Cariogram open access platform (https://cariogram.uni.mau.se/, accessed on 25.02.2026), an evidence-based digital tool designed to integrate multiple biological, behavioral, and clinical variables into a comprehensive, multifactorial caries risk profile. For the purposes of this study, the Cariogram was employed to contextualize salivary pH measurements within a broader preventive framework and to evaluate the contribution of salivary pH to the overall caries risk estimation. Data entered into the Cariogram included demographic information, dietary habits (frequency of meals and snacks and qualitative assessment of fermentable carbohydrate intake), oral hygiene status (objectively assessed using plaque disclosure), fluoride exposure (reported use of fluoride-containing toothpaste), previous caries experience, and salivary pH values. Dietary intake frequency was coded as the total number of daily meals and snacks; when a range was reported, the upper bound was retained for analysis, and when frequencies were reported as being below a given number, that number was assigned for coding purposes. Oral hygiene status was assessed clinically using the Silness–Löe Plaque Index and recorded as an ordinal variable (unsatisfactory, satisfactory, good, very good) for statistical analysis. Fluoride exposure was assessed based on the reported use of fluoridated toothpaste and recorded as a binary variable (yes/no). Based on these inputs, the software generated a graphical output representing the percentage probability of avoiding new carious lesions, along with categorical risk stratification (low, moderate, high, or very high). The Cariogram output was recorded for subsequent statistical analysis and used as the reference multifactorial risk-assessment framework within which digitally and colorimetrically determined salivary pH values were examined, in order to assess their clinical relevance and limitations as individual predictors.

### 2.3 Statistical analysis

Statistical analysis was performed using JASP software (version 0.95.4.0 for Windows). Continuous variables were initially explored for distributional characteristics using visual inspection of histograms and the Shapiro–Wilk test. Descriptive statistics were reported as mean ± standard deviation (SD) for normally distributed data and as median with interquartile range (IQR) for non-normally distributed data. Categorical and ordinal variables were summarized using absolute and relative frequencies.

Agreement between salivary pH measurements obtained using the digital device and pH indicator strips was evaluated using Bland–Altman analysis, with calculation of the mean difference (bias) and 95% limits of agreement. Confidence intervals (95% CI) were computed for both the mean difference and the limits of agreement. In addition, Spearman's rank correlation coefficient was used to evaluate the strength and direction of association between the two measurement methods.

Differences in salivary pH values according to saliva collection timing (morning vs. evening) were assessed using the Mann–Whitney *U* test for both digitally measured and strip-based pH values, as a robust non-parametric approach that does not require normality assumptions. Effect sizes were reported as rank-biserial correlation, as appropriate.

Associations between dietary intake frequency (total number of daily meals and snacks), salivary pH, and Cariogram-derived caries risk were explored using Spearman's rank correlation analysis, given the ordinal nature of the risk categories and the non-normal distribution of several variables. To evaluate the independent contribution of salivary pH within a multifactorial framework, a multivariable linear regression model was constructed using the Cariogram-derived chance to avoid new caries (%) as the dependent variable. Independent variables included mean digitally measured salivary pH, dietary intake frequency, oral hygiene status, use of fluoridated toothpaste, age, gender, and saliva collection timing (a.m./p.m.). Predictor selection was predefined on the basis of clinical relevance, established caries-related determinants, and the study objective of examining whether salivary pH contributed independently beyond behavioral and preventive factors. Mean digitally measured salivary pH was selected instead of strip-based pH because agreement analysis indicated greater precision and lower measurement error for the digital measurements. Model fit was assessed using overall model significance, *R*^2^, and adjusted *R*^2^. Regression assumptions were evaluated by inspection of residuals for linearity and homoscedasticity, assessment of approximate normality of residuals, and examination of multicollinearity using tolerance and variance inflation factor (VIF) values.

All variables required for the planned analyses were available for the 66 included participants. No missing data were identified for the primary outcome or for the independent variables included in the multivariable model; therefore, no imputation procedures were necessary.

A *p*-value <.05 was considered statistically significant.

For sample size estimation, an *a priori* power analysis was performed using G*Power v3.1 (Heinrich-Heine-Universität Düsseldorf, Düsseldorf, Germany) (18). Based on previously published pediatric data reporting a moderate-to-strong inverse association between salivary pH and caries indices (e.g., correlation magnitude around |*r*| ≈ 0.56) (19), we adopted a conservative expected effect size for planning. Assuming a two-tailed Pearson correlation, *α* = 0.05, and a target power of 85%, the required effect size was *r* = 0.36; therefore, the final sample size of 66 participants provides ≥85% power to detect correlations of *r* ≥ 0.36 between mean digitally measured salivary pH and the Cariogram outcome (20).

## 3 Results

### 3.1 Demographic and clinical characteristics of the study population

**Figure 1:**
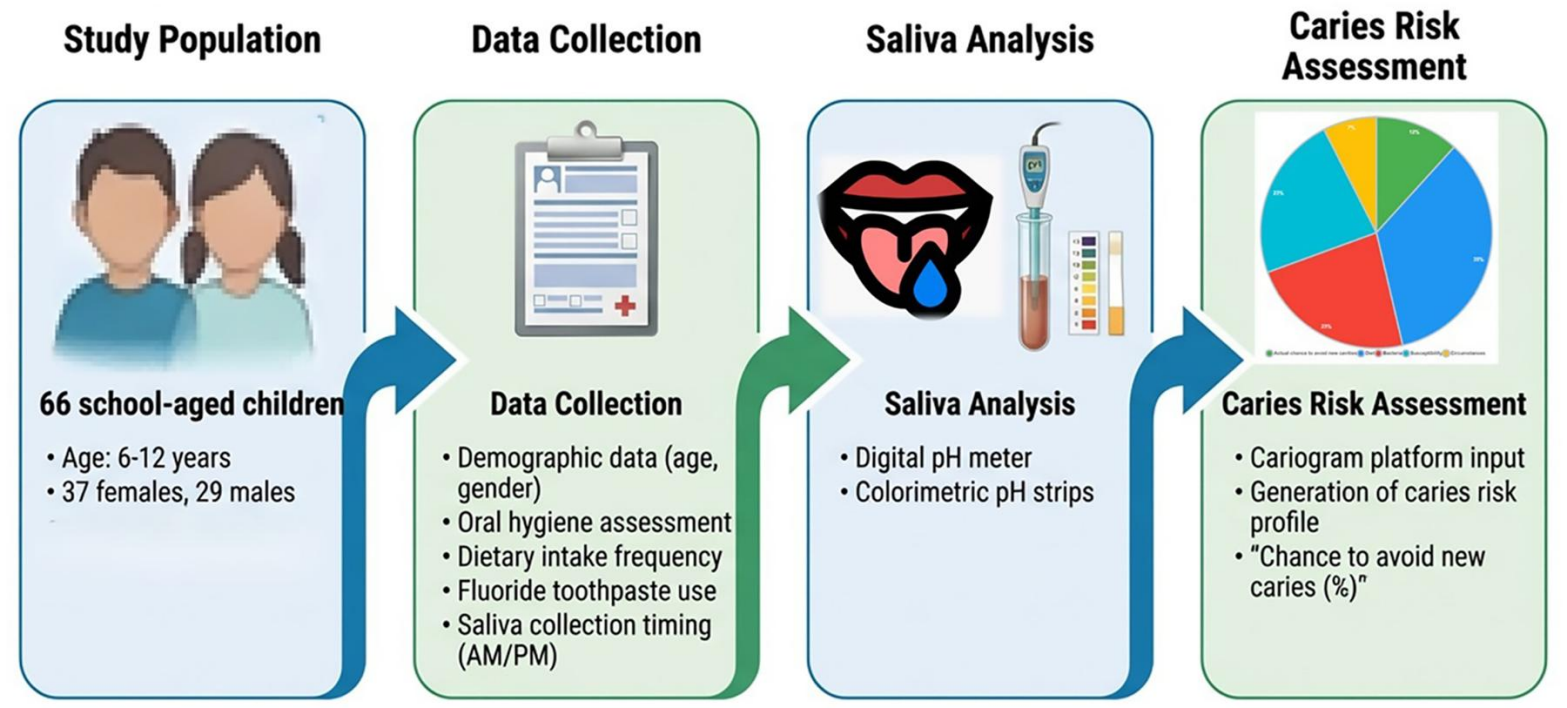
Included study population and assessment workflow. Demographic, behavioral, and clinical variables were recorded, salivary pH was measured using digital and strip-based methods, and caries risk was assessed using the Cariogram platform.

A total of 66 school-aged children, 37 girls (56.1%), mean (±SD) age 8.23 (±1.90) years old, were included in the analysis. The clinical, behavioral, salivary pH measurements and Cariogram-related characteristics of the study population are summarized in Table 1, and the assessment workflow is illustrated in Figure 1.

**Table 1:** Demographic, behavioral, clinical, salivary pH measurements, and Cariogram-related characteristics of the included pediatric cohort.

Variable	Value
Salivary pH measurements	mean ± SD [min–max]
Mean digital pH (a.m. + p.m.)	6.80 ± 0.27 (6.20–7.37)
Mean pH strip value (a.m. + p.m.)	6.57 ± 0.49 (5.82–7.33)
Dietary and preventive variables	
Daily meals and snacks, mean ± SD	5.49 ± 1.27
Oral hygiene status	*n* (%)
Unsatisfactory	8 (12.1)
Satisfactory	22 (33.3)
Good	28 (42.4)
Very good	8 (12.1)
Use of fluoride toothpaste	*n* (%)
Yes	61 (92.4)
No	5 (7.6)
Cariogram prognosis	
Cariogram caries risk category	*n* (%)
Low risk	8 (12.1)
Moderate risk	27 (40.9)
High risk	14 (21.2)
Very high risk	17 (25.8)
Chance to avoid new caries (%), mean ± SD	40.33 ± 18.35

Salivary pH values represent unstimulated saliva collected in the morning (a.m.) or evening (p.m.). Dietary intake frequency represents the total number of daily meals and snacks. Caries risk categories and chance to avoid new caries were derived using the Cariogram platform.

### 3.2 Associations between dietary intake, salivary pH, and caries risk

Spearman's rank correlation analysis revealed significant associations between dietary intake frequency, salivary pH—digitally assessed, and caries risk (Table 2). Higher daily meal and snack frequency was significantly associated with lower mean digitally measured salivary pH and with higher Cariogram-derived caries risk. A significant positive correlation was also observed between digitally measured and strip-based salivary pH values, indicating partial concordance between the two measurement methods. No other statistically significant correlations were identified.

**Table 2:** Bivariate associations between dietary intake frequency, salivary pH, and caries risk assessed using Spearman's rank correlation.

Variable	Daily meals/snacks	Mean digital pH	Mean strip pH	Cariogram risk
	Spearman's rho (*p*)	Spearman's rho (*p*)	Spearman's rho (*p*)	Spearman's rho (*p*)
Daily meals/snacks	—	**−0.381 (.002)** *	−0.017 (.894)	**0.442 (<.001)** *
Mean digital pH	**−0.381 (.002)** *	—	**0.278 (.024)** *	−0.126 (.314)
Mean strip pH	−0.017 (.894)	**0.278 (.024)** *	—	−0.116 (.355)
Cariogram risk	**0.442 (<.001)** *	−0.126 (.314)	−0.116 (.355)	—

* *p* < .05.

Bold values indicate statistically significant results (*p* < 0.05).

**Figure 2:**
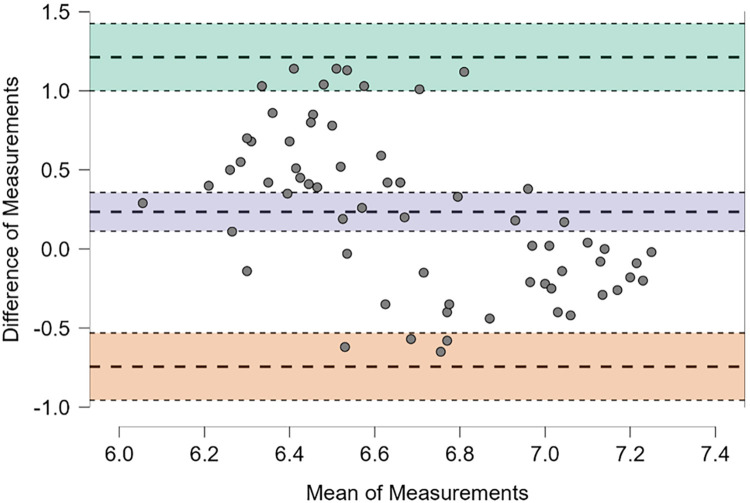
Bland–Altman plot illustrating agreement between mean digitally measured and mean strip-based salivary pH values. The mean difference (bias) was 0.24 (95% CI: 0.11–0.36). The 95% limits of agreement, calculated as bias ± 1.96 SD, ranged from −0.74 (95% CI: −0.96 to −0.53) to 1.00 (95% CI: 0.57–1.43). The bias and limits of agreement are indicated by distinct colored horizontal lines in the plot.

A weak but statistically significant positive association was observed between digitally measured and strip-based salivary pH values (Spearman's rho = 0.278, *p* = 0.024). However, correlation analysis alone does not assess agreement between measurement methods. Consistent with this, Bland–Altman analysis (Figure 2) demonstrated a small positive mean bias of 0.24 pH units, indicating that digitally measured values tended to be slightly higher than strip-based measurements. The 95% limits of agreement were relatively wide (−0.74 to 1.00 pH units), indicating substantial interindividual variability between methods despite statistical correlation. Together, these findings suggest partial concordance but limited interchangeability between digital and strip-based salivary pH measurements.

No statistically significant differences in salivary pH values were observed between morning and evening collection times for either digitally measured or strip-based pH (Mann–Whitney *U* test, *p* > .05 for both). Effect size estimates were negligible, indicating no clinically relevant timing effect.

### 3.3 Multivariable analysis of factors associated with caries risk

A multivariable linear regression model was constructed using the Cariogram-derived chance to avoid new caries (%) as the dependent variable. The model included mean digitally measured salivary pH, daily meal and snack frequency, age, gender, saliva collection timing (a.m./p.m.), use of fluoridated toothpaste, and oral hygiene status. Mean digitally measured salivary pH was selected for inclusion in the multivariable model because agreement analysis demonstrated a systematic bias and greater variability for strip-based measurements, while digital pH values showed higher precision and reduced measurement error, thereby providing a more reliable continuous predictor for regression analysis.

After adjustment for all covariates, dietary intake frequency, saliva collection timing, use of fluoridated toothpaste, and oral hygiene status were independently associated with the Cariogram-derived chance to avoid new caries (Table 3).

**Table 3:** Multivariable linear regression model assessing the independent association of salivary pH, dietary intake frequency, oral hygiene status, fluoride toothpaste use, and demographic variables with Cariogram-derived chance to avoid new caries (%).

Predictor	*B*	95% CI	*p* value
Mean digital pH	−0.169	−0.832 to 0.494	.611
Daily meal/snack frequency	0.192	0.036 to 0.347	**.** **016** *
Age	−0.068	−0.165 to 0.029	.167
Gender (male)	0.155	−0.166 to 0.475	.339
Timing (p.m. vs. a.m.)	−0.500	−0.825 to −0.175	**.** **003** *
Fluoridated toothpaste (yes)	−0.746	−1.355 to −0.138	**.** **017** *
Oral hygiene—satisfactory	−0.489	−1.028 to 0.049	.074
Oral hygiene—good	−1.182	−1.687 to −0.676	**<.001** *
Oral hygiene—very good	−2.144	−2.801 to −1.488	**<.001** *

Model summary: *R*^2^ = 0.669; adjusted *R*^2^ = 0.616; *F*_(9,56)_ = 12.55.

* *p* < .05.

Bold values indicate statistically significant results (*p* < 0.05).

A higher daily frequency of meals and snacks was significantly associated with the fitted outcome (*B* = 0.192, 95% CI: 0.036–0.347; *p* = .016). Sampling during the p.m. session, compared with the a.m. session, was associated with the fitted outcome (*B* = −0.500, 95% CI: −0.825 to −0.175; *p* = .003). Use of fluoridated toothpaste was also associated with the fitted outcome (*B* = −0.746, 95% CI: −1.355 to −0.138; *p* = .017).

Oral hygiene status showed a dose–response relationship with the Cariogram-derived chance to avoid new caries. Compared with the reference category, progressively better oral hygiene was associated with a higher chance of avoiding new caries, with the strongest protective effect observed for very good oral hygiene (*B* = −2.144, 95% CI: −2.801 to −1.488; *p* < .001).

In contrast, mean digitally measured salivary pH was not independently associated with the Cariogram-derived chance to avoid new caries, after adjustment for dietary and behavioral factors (*B* = −0.169, 95% CI: −0.832 to 0.494; *p* = 0.611). Age and gender were also not significant predictors in the adjusted model (*p* > .05).

The full model demonstrated strong overall explanatory power, accounting for 66.9% of the variance in the dependent variable (*R*^2^ = 0.669; adjusted *R*^2^ = 0.616). The model was statistically significant [*F*_(9,56)_ = 12.55, *p* < .001], indicating that the included predictors jointly contributed to variation in the fitted outcome. No evidence of problematic multicollinearity was observed (all VIF values <1.3). Inspection of model diagnostics did not indicate major violations of the regression assumptions.

## 4 Discussion

The present study aimed to characterize caries risk profiles in a school-aged pediatric cohort using the Cariogram platform as a structured multifactorial caries risk-assessment tool and to examine whether digitally measured salivary pH contributed independently to risk stratification within this framework.

The novelty of the present study lies in its integrated evaluation of salivary pH as a non-invasive biomarker within an established multifactorial caries risk-assessment framework implemented using the Cariogram platform, together with digital pH measurement in a pediatric population (15). Unlike previous studies that have examined salivary pH either as an isolated parameter or within limited risk models, this investigation simultaneously addressed the methodological rigor of salivary pH determination and its potential adjunctive clinical value when interpreted within a structured multifactorial risk-assessment platform (Cariogram). The results directly address these objectives and support the contemporary view that salivary pH, although biologically relevant, does not act as an independent predictor of caries risk when behavioral and preventive factors are simultaneously considered (11, 12, 15).

The distribution of digitally measured salivary pH values (6.2–7.4), with a predominant peak between 6.6 and 6.8, reflects the basal oral environment of a pediatric population enrolled in a preventive care program. Similar ranges have been reported in children receiving regular prophylaxis and fluoride exposure, suggesting a relatively stable oral homeostasis under preventive conditions (21, 22). Nevertheless, this range remains mildly acidic and may become clinically relevant in the presence of frequent fermentable carbohydrate intake, persistent dental biofilm, or inadequate brushing technique, even when fluoride toothpaste use is reported (11, 23–25).

From a methodological standpoint, the observed mean difference of approximately 0.24 pH units between digital measurements and colorimetric strips confirms previous reports that strip-based methods tend to underestimate salivary pH and are subject to observer-dependent variability (15, 26, 27). This systematic bias is particularly relevant near interpretative thresholds (e.g., 6.5–6.8), where small numerical differences may influence clinical judgment. While colorimetric strips may remain useful for large-scale screening or educational purposes, digital pH-metry provides superior precision and reproducibility for research settings, longitudinal monitoring, and individualized preventive decision-making (28).

Despite its limitations, salivary pH testing remains justified within caries risk assessment due to its practical accessibility and minimal technical requirements. Digital salivary pH meters are relatively inexpensive, widely available, and allow immediate chairside assessment without the need for laboratory processing or specialized consumables. These characteristics make pH-meters particularly suitable for preventive dental settings and community-oriented programs. However, practical constraints must be acknowledged, especially in pediatric populations. The requirement for a minimum volume of unstimulated saliva may limit feasibility in younger children or in those with reduced cooperation, and collection variability may influence sample adequacy. These considerations further support the interpretation of salivary pH not as a standalone diagnostic marker, but as a contextual biological parameter within a multifactorial caries risk model. When integrated alongside dietary habits, oral hygiene status, and fluoride exposure—as implemented in the Cariogram framework—salivary pH may provide supportive clinical context for tailoring preventive recommendations, such as adjusting meal frequency, reinforcing toothbrushing routines, and individualizing prophylactic recall intervals.

A central finding of this study is the very weak association between basal salivary pH and Cariogram-derived caries risk, together with the lack of an independent contribution of pH in the multivariable model. This result is biologically and methodologically plausible and aligns with current understanding of caries pathogenesis. Dental caries is not driven by a single static parameter, but by the cumulative effect of repeated acid challenges generated within the dental biofilm following fermentable carbohydrate intake (24, 25). Basal salivary pH represents only a snapshot of oral ecology and does not capture critical dynamic processes, such as the magnitude of postprandial pH drop (Stephan curve), the duration spent below the critical pH threshold, the frequency of acidogenic episodes, or the effective buffering capacity of saliva (21, 29). Consequently, a child may present with a “normal” resting pH while still exhibiting a high caries risk in the context of frequent snacking, visible plaque accumulation, and suboptimal fluoride exposure.

Within this context, the present findings reinforce the conceptual foundation of the Cariogram platform, which integrates biological, behavioral, and clinical variables into a composite risk estimate rather than relying on isolated markers (5, 30, 31). The distribution of caries risk categories observed in this cohort—despite regular prophylactic monitoring—indicates that a substantial proportion of children remain within moderate to high-risk strata, a finding consistent with previous pediatric validation studies of Cariogram (30, 32). The weak contribution of salivary pH to the final risk score does not undermine the validity of Cariogram-based stratification; instead, it confirms that behavioral determinants and biofilm control dominate caries risk, while individual salivary parameters exert limited standalone influence within a multifactorial model.

An additional dimension relevant to pediatric salivary diagnostics is the psychophysiological component. Dental anxiety and anticipatory stress have been shown to influence salivary parameters through transient reductions in salivary flow rate and buffering capacity, potentially affecting pH measurements obtained in the clinical setting (27, 33). In this regard, salivary pH may partially reflect acute emotional states in addition to habitual oral conditions, supporting its interpretation as an interface biomarker linking oral ecology and pediatric psychophysiology rather than a purely dietary marker.

From a clinical perspective, these findings support the integration of digital salivary pH measurement as a chairside tool in pediatric preventive dentistry, not as a singular diagnostic criterion but as an adjunctive and educational parameter. Objective numerical feedback may enhance parental understanding of the relationship between dietary habits, oral hygiene, and caries risk. The Cariogram platform remains valuable for recall stratification and justification of individualized preventive strategies, whereas exclusive reliance on colorimetric strips—particularly in borderline cases—should be avoided due to systematic underestimation (26).

Looking ahead, the results align with current trends toward digitalization and the application of artificial intelligence (AI) in caries prevention. Digital pH-meters provides objective and repeatable biological data, while Cariogram offers a validated multifactorial framework; AI represents the analytical layer capable of identifying longitudinal patterns across repeated pH measurements, behavioral data, stress-related variables, and preventive compliance (15, 34, 35). The development of parent-oriented mobile health applications integrating salivary biomarkers, dietary and hygiene questionnaires, automated recommendations, reminders, and clinician–family communication represents a realistic pathway toward personalized and preventive oral healthcare (36–38).

Several limitations should be considered when interpreting these findings. The cross-sectional design precludes causal inference and does not capture postprandial salivary pH dynamics, while single-point basal pH measurements do not reflect Stephan curve behavior or the duration of time spent below critical demineralization thresholds. In addition, because saliva collection took place during either morning or evening clinical sessions according to appointment availability rather than random allocation, some residual circadian or scheduling-related measurement bias cannot be excluded, even though pre-analytical restrictions were applied and no significant univariable differences in measured salivary pH were observed between a.m. and p.m. sessions. The absence of microbiological assessments and direct buffering-capacity measurements further limits the biological resolution of the analysis. Because the study cohort consisted of prophylactically monitored urban children, the findings may not be fully generalizable to underserved or higher-risk pediatric populations. A further methodological limitation relates to the multivariable analysis: although the regression model was specified *a priori* on the basis of clinical relevance, the sample size was relatively modest in relation to the number of modeled parameters, particularly because oral hygiene status required multiple indicator terms. In addition, because the regression outcome was derived from the Cariogram algorithm and several covariates overlapped with Cariogram inputs, some degree of structural dependency cannot be excluded. Consequently, some regression coefficients may be unstable, and the model may be susceptible to overfitting despite the absence of problematic multicollinearity. The multivariable findings should therefore be regarded as exploratory and confirmed in larger, independent pediatric cohorts. Overall, these limitations reinforce the exploratory nature of the study and highlight the need for future longitudinal investigations incorporating repeated salivary pH measurements, early lesion incidence, and additional salivary biomarkers, including buffering capacity, inflammatory markers, and microbiological profiles (39).

Within these constraints, the present findings indicate that salivary pH is a biologically relevant but insufficient standalone marker, whose clinical relevance is best understood when interpreted within an established multifactorial caries risk-assessment strategy supported by digital tools.

## 5 Conclusion

Within the limitations of this study, salivary pH appears to be a biologically relevant but non-independent indicator of caries risk in school-aged children when interpreted within a multifactorial caries risk-assessment framework. Digitally measured salivary pH reflected a relatively stable basal oral environment in this prophylactically monitored pediatric cohort, but it showed limited ability to discriminate between caries risk categories when behavioral and preventive factors were considered simultaneously.

Comparison of digital and strip-based pH measurements demonstrated systematic bias and greater variability for colorimetric strips, supporting the use of digital pH measurement as the more precise and reproducible approach for research and longitudinal follow-up. However, the present study does not propose a novel digital model; rather, it supports the clinical utility of evaluating salivary pH within an existing structured framework such as the Cariogram platform.

Multivariable analysis confirmed that dietary intake frequency, oral hygiene status, fluoride toothpaste use, and contextual factors were more strongly associated with caries risk than basal salivary pH. Accordingly, salivary pH should be regarded as an adjunctive biomarker that may support patient education and preventive decision-making when used alongside established multifactorial risk assessment, rather than as a standalone diagnostic criterion.

## Data Availability

The raw data supporting the conclusions of this article will be made available by the authors, without undue reservation.

## References

[1] Cheever VJ Mohajeri A Patel K Burris RC Hung M. Impact of free sugar consumption on dental caries: a cross-sectional analysis of children in the United States. Dent J. (2025) 13(2):48. 10.3390/DJ13020048/S1 PMC11854531 39996922

[2] Giacaman RA Fernández CE Sandoval CM León S Manríquez NG Echeverría C Understanding dental caries as a non-communicable and behavioral disease: management implications. Front Oral Health. (2022) 3:764479. 10.3389/FROH.2022.764479 36092137 PMC9448953

[3] Lynch RJM. The primary and mixed dentition, post-eruptive enamel maturation and dental caries: a review. Int Dent J. (2013) 63(Suppl 2):3–13. 10.1111/IDJ.12076 24283279 PMC9375027

[4] Han SY Chang CL Wang YL Wang CS Lee WJ Vo TTT A narrative review on advancing pediatric oral health: comprehensive strategies for the prevention and management of dental challenges in children. Children. (2025) 12(3):286. 10.3390/CHILDREN12030286 40150569 PMC11941194

[5] Ng TCH Luo BW Lam WYH Baysan A Chu CH Yu OY. Updates on caries risk assessment—a literature review. Dent J. (2024) 12(10):312. 10.3390/DJ12100312 PMC11506515 39452440

[6] Ismail AI Pitts NB Tellez M Banerjee A Deery C Douglas G The international caries classification and management system (ICCMS™) an example of a caries management pathway. BMC Oral Health. (2015) 15(Suppl 1):S9. 10.1186/1472-6831-15-S1-S9 26391116 PMC4580843

[7] Maheswari SU Raja J Kumar A Seelan RG. Caries management by risk assessment: a review on current strategies for caries prevention and management. J Pharm Bioallied Sci. (2015) 7(Suppl 2):S320–4. 10.4103/0975-7406.163436 26538870 PMC4606612

[8] International Caries Classification and Management System (ICCMS). Available online at: https://www.iccms-web.com/ (Accessed January 2, 2026).

[9] Hegde MN Attavar SH Shetty N Hegde ND Hegde NN. Saliva as a biomarker for dental caries: a systematic review. J Conserv Dent. (2019) 22(1):2. 10.4103/JCD.JCD_531_18 30820074 PMC6385571

[10] Enax J Fandrich P Schulze Zur Wiesche ES Epple M Enax J Fandrich P The remineralization of enamel from saliva: a chemical perspective. Dent J. (2024) 12(11):339. 10.3390/DJ12110339 PMC11592461 39590389

[11] Delvalle CSG Vivó JC Reichard G Simon LP del Cojo MB Pérez EMM Caries index and salivary factors in children: a case–control study. Children. (2025) 12(12):1631. 10.3390/CHILDREN12121631 41462771 PMC12731405

[12] Kuri PN Thimmegowda U. Assessment of salivary pH, flow rate, buffering capacity and alpha-amylase enzyme activity in caries-free and caries-active children: a cross-sectional study. J Clin Diagn Res. (2025) 19(9). 10.7860/JCDR/2025/76380.2144940928211

[13] Putri R Fadilah N Rivmawati L Nawawi AP Supriatna A Pribadi AP Correlation analysis of saliva volume and salivary pH on dental caries status in children aged 11–12 years using the HI BOGI application: a cross sectional study. Padjadjaran J Dent. (2025) 37(3):382–93. 10.24198/PJD.VOL37NO3.66132

[14] Çiftçi BT Aşantoğrol F. Utilization of machine learning models in predicting caries risk groups and oral health-related risk factors in adults. BMC Oral Health. (2024) 24(1):430. 10.1186/S12903-024-04210-Z/FIGURES/14 38589865 PMC11000438

[15] Sgiea ED Cristache CM Mihut T Drafta S Beuran IA. The integration of salivary pH meters and artificial intelligence in the early diagnosis and management of dental caries in pediatric dentistry: a scoping review. Oral. (2025) 5(1):12. 10.3390/ORAL5010012/S1

[16] Bhatia S Gupta VK Kumar S Mishra G Malhotra S Arif K Artificial intelligence based techniques for caries risk prediction and assessment: a scoping review. J Oral Biol Craniofac Res. (2025) 15(6):1497–507. 10.1016/J.JOBCR.2025.08.027 40995586 PMC12455132

[17] American Academy of Pediatric Dentistry. Caries-risk assessment and management for infants, children, and adolescents. Pediatr Dent. (2017) 39(6):197–204. 29179357

[18] Erdfelder E FAul F Buchner A Lang AG. Statistical power analyses using G*power 3.1: tests for correlation and regression analyses. Behav Res Methods. (2009) 41(4):1149–60. 10.3758/BRM.41.4.1149/METRICS 19897823

[19] Basch Y Peretz B. Salivary pH levels and caries among siblings and parents within families. J Clin Pediatr Dent. (2013) 38(2):129–32. 10.17796/JCPD.38.2.90764500T2703188 24683775

[20] Kang H. Sample size determination and power analysis using the G*power software. J Educ Eval Health Prof. (2021) 18:17. 10.3352/JEEHP.2021.18.17 34325496 PMC8441096

[21] Dawes C. What is the critical pH and why does a tooth dissolve in acid? J Can Dent Assoc. (2003) 69(11):722–4. 14653937

[22] Antonelli R Massei V Ferrari E Gallo M Pertinhez TA Vescovi P Salivary diagnosis of dental caries: a systematic review. Curr Issues Mol Biol. (2024) 46(5):4234–50. 10.3390/CIMB46050258/S1 38785526 PMC11120503

[23] Moynihan PJ. Dietary advice in dental practice. Br Dent J. (2002) 193(10):563–8. 10.1038/SJ.BDJ.4801628 12481178

[24] Moynihan PJ Kelly SAM. Effect on caries of restricting sugars intake: systematic review to inform WHO guidelines. J Dent Res. (2014) 93(1):8–18. 10.1177/0022034513508954 24323509 PMC3872848

[25] Moynihan PJ Kelly SAM. Sugar and dental caries: a reappraisal of the quantitative relationship between sugar intake and dental caries. Br Dent J. (2014) 217(9):525. 10.1038/sj.bdj.2014.976

[26] Shamsi-Basha B Bernard-Garbati R Mortier E Grasland A Lachgar K Alantar A. Evidence-based practice for the use of pH indicator paper strip in oral medicine: a literature review. Cureus. (2024) 16(6):e62797. 10.7759/CUREUS.62797 39040739 PMC11260637

[27] Sgîea ED Mihuţ T Văcărel EV Cristache CM. The influence of dental anxiety on salivary pH in children: a pilot study. Dent Target. (2025) 20(1):31–4.

[28] González-González M Flores-Dela Toba R Ortiz-Martínez M Rito-Palomares M. Development and evaluation of colorimetric pH determination methods as a potential tool for biomarkers monitoring. J Chem Technol Biotechnol. (2025). 10.1002/jctb.7900

[29] Catunda RQ Altabtbaei K Flores-Mir C Febbraio M. Pre-treatment oral microbiome analysis and salivary Stephan curve kinetics in white spot lesion development in orthodontic patients wearing fixed appliances. A pilot study. BMC Oral Health. (2023) 23(1):239. 10.1186/S12903-023-02917-Z 37095478 PMC10127078

[30] Campus G Cagetti MG Sale S Carta G Lingström P. Cariogram validity in schoolchildren: a two-year follow-up study. Caries Res. (2012) 46(1):16–22. 10.1159/000334932 22222621

[31] Paul P Kalghatgi S Dalvi T Magdum SS Paul P Kalghatgi S Advancing dental risk profiling: a literature review of the Cariogram model. Cureus. (2025) 17(3):e80069. 10.7759/CUREUS.80069 40190924 PMC11969287

[32] Dou L Luo J Fu X Tang Y Gao J Yang D. The validity of caries risk assessment in young adults with past caries experience using a screening Cariogram model without saliva tests. Int Dent J. (2018) 68(4):221–6. 10.1111/IDJ.12378 29572813 PMC9378905

[33] Main S Suchy S Carrilho M Sharmin Z Hall S Rao J A cross-sectional study of salivary cortisol, alpha amylase, and measures of psychological distress in children undergoing dental procedures. Children. (2025) 12(9):1235. 10.3390/CHILDREN12091235/S1 41007100 PMC12468251

[34] Schwendicke F Samek W Krois J. Artificial intelligence in dentistry: chances and challenges. J Dent Res. (2020) 99(7):769–74. 10.1177/0022034520915714 32315260 PMC7309354

[35] Dettori M Lamloum D Lingström P Campus G. Artificial intelligence and innovation in oral health care sciences: a conceptual review. Healthcare. (2025) 13(24):3327. 10.3390/HEALTHCARE13243327/S1 41464395 PMC12733269

[36] Liang Y Li D Deng D Chu CH Mei ML Li Y AI-driven dental caries management strategies: from clinical practice to professional education and public self care. Int Dent J. (2025) 75(4):100827. 10.1016/J.IDENTJ.2025.04.007 40354695 PMC12138923

[37] Murariu A Bobu L Gelețu GL Stoleriu S Iovan G Vasluianu RI The impact of mobile applications on improving oral hygiene knowledge and skills of adolescents: a scoping review. J Clin Med. (2025) 14(9):2907. 10.3390/JCM14092907/S1 40363939 PMC12072554

[38] Vacarel EV Barbulescu ED Cristache CM. From saliva to diagnosis: a scoping review of conventional and biosensor-based methods for salivary biomarkers in chronic kidney disease. Diagnostics. (2025) 15(17):2226. 10.3390/DIAGNOSTICS15172226 40941713 PMC12427769

[39] Xu S Mumuni AN Tuason RTS Maki KA. Methodological considerations in saliva-based biomarker research: addressing patient-specific variability in translational research protocols. Curr Protoc. (2025) 5(10):e70235. 10.1002/CPZ1.70235 41148055 PMC12560811

